# Nitrite and Nitrate Levels in Follicular Fluid From Human Oocyte Donors Are Related to Ovarian Response and Embryo Quality

**DOI:** 10.3389/fcell.2021.647002

**Published:** 2021-04-14

**Authors:** Florentin-Daniel Staicu, Analuce Canha-Gouveia, Cristina Soriano-Úbeda, Juan Carlos Martínez-Soto, Evdochia Adoamnei, Jorge E. Chavarro, Carmen Matás

**Affiliations:** ^1^Department of Physiology, Faculty of Veterinary Science, International Excellence Campus for Higher Education and Research (Campus Mare Nostrum), University of Murcia, Murcia, Spain; ^2^Biomedical Research Institute of Murcia (IMIB), Murcia, Spain; ^3^Department of Veterinary and Animal Sciences, University of Massachusetts Amherst, Amherst, MA, United States; ^4^IVI-RMA Global, Murcia, Spain; ^5^Department of Nursing, School of Nursing, University of Murcia, Murcia, Spain; ^6^Department of Nutrition, Harvard T.H. Chan School of Public Health, Boston, MA, United States; ^7^Department of Epidemiology, Harvard T.H. Chan School of Public Health, Boston, MA, United States; ^8^Channing Division of Network Medicine, Brigham and Women’s Hospital, Harvard Medical School, Boston, MA, United States

**Keywords:** nitric oxide, nitrite, nitrate, follicular fluid, oocyte, embryo

## Abstract

Nitric oxide, a key regulatory molecule in the follicular fluid, has been suggested as a possible biomarker to predict ovarian response in stimulated cycles and the potential of the retrieved oocytes for developing high-quality embryos. Nevertheless, a consensus on whether or not nitric oxide can help in this context has not been reached. We simultaneously measured the oxidation products of nitric oxide, nitrite, and nitrate, via high-performance liquid chromatography (HPLC)-UV in follicular fluid samples from 72 oocyte donors. We found no associations of follicular fluid nitrite, nitrate, total nitric oxide, or nitrate/nitrite ratio with total or metaphase II (MII) oocyte yield. However, nitrite and nitrate levels were related to the yield of MII oocytes when this outcome was expressed as a proportion of all oocytes retrieved. The adjusted MII proportion in the lowest and highest nitrite levels were 68% (58–77%) and 79% (70–85%), respectively (p, linear trend = 0.02), whereas the adjusted MII proportion in extreme tertiles of nitrate levels were 79% (70–85%) and 68% (57–77%) (p, linear trend = 0.03). In addition, nitrate levels showed a suggestive inverse correlation with embryos with maximum or high potential of implantation (*p* = 0.07). These results suggest that the follicular fluid concentrations of nitrite and nitrate may be a useful tool in predicting how healthy oocyte donors respond to superovulation and the implantation potential of the embryos produced from their oocytes.

## Introduction

A large number of couples of reproductive age struggle with infertility issues, the causes of which are not always clear (Agarwal and Allamaneni, 2004). For this reason, several studies have tried to identify new biochemical markers that can affect gamete and embryo quality and may predict the outcome of infertility treatment with *in vitro* fertilization (IVF) (Yalçınkaya et al., 2013).

Nitric oxide (NO) has emerged as a candidate predictor of ovarian response and IVF outcomes (Barroso et al., 1999). Apart from being a well-known regulator of vasodilation and neurotransmission (Pacher et al., 2007), NO has also been linked to the granulosa cell function (Basini et al., 1998), meiotic resumption (Romero-Aguirregomezcorta et al., 2014), and prevention of oocyte aging (Goud et al., 2005), as well as ovulation (Anteby et al., 1996). However, when investigating the relation of intrafollicular levels of NO with oocyte recruiting, fertilization potential, embryo quality, implantation, and pregnancy rates in patients undergoing IVF, the results are contradictory. On one hand, evidence suggests that NO levels in the follicular fluid (FF) are inversely associated with the fertilization of mature oocytes and the ability of the subsequent embryo to cleave normally (Barrionuevo et al., 2000). Furthermore, a negative correlation between FF NO levels and embryo morphology was identified (Barroso et al., 1999; Battaglia et al., 2006). On the other hand, other studies found no differences in relation to oocyte and embryo quality (Lee et al., 2000; Yalçınkaya et al., 2013). The relationship between FF, NO, and pregnancy outcome is also uncertain (Lee et al., 2000; Kim et al., 2004; Yalçınkaya et al., 2013).

The controversy among these data might reside in the unstable nature of NO, which makes its direct measurement difficult (Manau et al., 2000). NO has a short biological half-life that can be influenced by different factors, such as its concentration, the presence or not of NO scavengers (Hakim et al., 1996), and the cellular redox state (Rosselli et al., 1998). NO itself can act as a free radical scavenger and prevent cell toxicity by inactivating the superoxide anion. Under specific conditions, however, this interaction can generate peroxynitrite, a potent oxidant (Rosselli et al., 1998). Additionally, NO is oxidized in blood and tissues leading to the formation of two stable end-products, nitrite (NO_2_) and nitrate (NO_3_) (Lundberg et al., 2008), which are a suitable to quantify indirectly NO synthesis (Romitelli et al., 2007). However, the methods for NO_2_ and NO_3_ detection have limitations such as the time required for the analysis, interference from other ions, or the difficulty to detect NO_2_ and NO_3_ at the same time and in minor concentrations (Croitoru, 2012). The simultaneous detection of low NO_2_ and NO_3_ concentrations has been described in mammal blood, urine, and in plant samples by high-performance liquid chromatography (HPLC)-UV (Croitoru, 2012). This study aims, first of all, to apply this method with some modifications to determine the FF levels of NO_2_, NO_3_, total NO (NOx), and NO_3_/NO_2_ ratio in oocyte donors. Subsequently, it aims to further clarify any associations between these parameters, ovarian response, and quality of the embryos derived from oocytes of healthy women undergoing ovarian stimulation as part of their participation in an oocyte donation program.

## Materials and Methods

### Ethics

This study was approved by the Ethics Review Committee of CEIC Hospital General Universitario Jose Maria Morales Meseguer (Murcia, Spain) (Approval No. EST: 06/17) and registered at https://clinicaltrials.gov/ (ID: NCT03307655). Women participating in the gamete donation program at IVI-RMA Global (Murcia, Spain) were invited to participate in the study. All the donors who accepted provided their written informed consent.

### Clinical and Lifestyle-Related Data

The following data regarding the donation cycle were collected from IVI-RMA Global (Murcia, Spain) for all participants: total and metaphase II (MII) oocyte yield, number of stimulation days, 17β-estradiol (E2) peak, total dose of follicle-stimulating hormone (FSH), oocyte fate (fresh transfer, vitrified and mixed), and quality of the blastocyst derived from their oocytes. Donors who enrolled in the study were asked to report their demographic and lifestyle characteristics over the previous year such as age, body mass index (BMI), sleep time, diet, coffee and alcohol consumption, smoking history, leisure physical activity, and sedentary behavior. The smoking history was classified in three different categories (ever smoker, self-reported exposure to secondhand smoke) and physical activity in Leisure time vigorous/moderate physical activities, hours/week, and Sedentary behavior hours/week. BMI was calculated as weight (kg) divided by height squared (m^2^). The sleep was evaluated distinguishing the hours of sleep per day and the napping time. The mean number of servings per week of several food items was recorded using a food frequency questionnaire adapted to meet specific study objectives. Special attention was given to specific vegetables known by its high mean nitrate content, like spinach (Lidder and Webb, 2013). Later, according to Panagiotakos et al. (2007), all the listed food items were rearranged in different groups presumed to be close to Mediterranean dietary pattern, namely, the non-refined cereals, fruits, vegetables, legumes, olive oil, fish, and potatoes group. For example, the vegetables group was formed by the sum of cabbage, cauliflower, broccoli, cooked or raw, artichokes, asparagus, carrot, spinach or cooked chard, lettuce, endives, onion, cooked green beans, aubergines, zucchini, cucumbers, peppers, boiled corn, and tomato. A different score scale (0–5 for never, rare, frequent, very frequent, weekly, and daily consumption) was assigned to each group. Subsequently, the Mediterranean diet score (on a scale of 0–55) was calculated by summing up the corresponding scores of these groups. To calculate the total intake, the alcohol content for specific items was summed and multiplied by weights proportional to the frequency of use of each item.

### Superovulation of Oocyte Donors and Oocyte Retrieval

Superovulation was achieved by means of a human recombinant follicle-stimulating hormone and a gonadotrophin-releasing hormone antagonist, as previously described (Melo et al., 2009). When the follicles reached an average diameter of 17.5–18 mm, a gonadotropin-releasing hormone (GnRH) agonist was administered to induce ovulation (Melo et al., 2009). Approximately 36 h later, dominant follicles were punctured transvaginally under ultrasound guidance, and FF was aspirated together with the oocyte in Sequential Fert^TM^ medium (Origio^®^, CooperSurgical Fertility and Genomic Solutions, Målov, Denmark).

### Intracytoplasmic Sperm Injection and Embryo Culture

**FIGURE 1:**
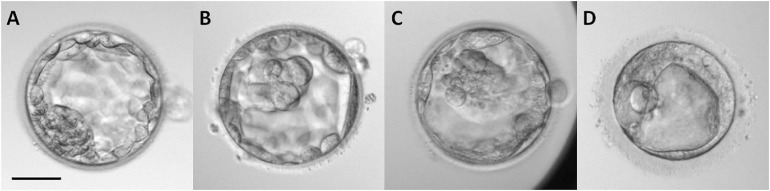
Morphological classification of blastocysts in day 5 of *in vitro* development according to the criteria established by the Spanish Association for the Study of the Biology of Reproduction (ASEBIR) based on the morphological evaluation of the inner cell mass (ICM) and trophectoderm (TE) (Hurtado de Mendoza et al., 2015; Cuevas Saiz et al., 2018). The implantation potential of blastocysts according to this classification is established as follows: grade **(A)**, maximum; grade **(B)**, high; grade **(C)**, medium; grade **(D)**, low (see Table 1). Scale bar represents 25 μm.

After retrieval, the oocytes of 72 donors were washed in Sequential Fert^TM^ medium. The removal of cumulus cells was performed by gently pipetting the oocytes in a solution of 80 IU/ml hyaluronidase in Sequential Fert^TM^ medium. Oocytes were cultured in fertilization medium (Gems^®^, Genea Biomedx, Sydney, NSW, Australia) at 5% CO_2_, 37°C, and atmospheric O_2_ for 3 h. After that, the oocytes were placed in a microdrop of fertilization medium for performing intracytoplasmic sperm injection (ICSI). Sperm samples were obtained from ejaculates without oligo-asteno-teratozoospermia and selected by density gradient 45/90% (SIP100, Sil-Select Plus, FERTIPRO NV, Belgium), diluted in a solution of 10% polyvinylpyrrolidone (PVP) in Sequential Fert^TM^ medium, and placed in a microdrop for performing ICSI. Immediately after ICSI, the zygotes were transferred to the pre-equilibrated embryo culture medium Cleavage Medium^TM^ (Gems^®^, Genea Biomedx, Sydney, NSW, Australia) and covered with mineral oil (LifeGuard^®^, Genomicks Sdn Bhd, Petaling Jaya, Malaysia) and cultured for 5 days. Several parameters were analyzed in the embryos: the fertilization rate (%), embryo rate (%) – percentage of embryos that cleaved – and quality of the embryos at day 5. Embryo quality was evaluated by morphological scoring at day 5 of culture (blastocyst stage) by experienced technicians according to the standardized criteria of the Spanish Association for the Study of the Biology of Reproduction (ASEBIR) (Hurtado de Mendoza et al., 2015; Cuevas Saiz et al., 2018; Figure 1). The morphological parameters considered for this evaluation were the size and cellular cohesion of the blastomeres in the inner cell mass (ICM) and the homogeneity, cohesion, and number of cells of the trophectoderm (TE). According to these criteria, embryos were classified in four grades (A–D) summarized in Table 1. Degenerated or dead oocytes and embryos were excluded from the study. The retrieved oocytes of 72 donors were used for one respective recipient. However, since three of those 72 donors had donated an elevated amount of MII oocytes, their oocytes were used for more than one recipient and, consequently, were inseminated with spermatozoa of different men. Therefore, we present a total *n* = 75 of embryo data.

**TABLE 1:** Spanish Association for the Study of the Biology of Reproduction (ASEBIR) scoring for blastocyst stage in day 5 of embryo development according to morphological evaluation of the inner cell mass (ICM) and trophectoderm (TE) (Hurtado de Mendoza et al., 2015; Cuevas Saiz et al., 2018).

	**Inner cell mass (ICM)**		**Trophectoderm (TE)**	**Embryo quality**	**Potential of implantation**
**Embryo grade**	**Blastomeres size**	**Cellular cohesion**			
A	3800–1900 μm^2^	Compacted	Homogeneous, cohesive, many cells	1st or optimus	Maximum
B	3800–1900 μm^2^	Loose	Homogeneous, not many cells	2nd or good	High
C	1900 μm^2^	Any	Few cells	3rd or medium	Medium
D	Ongoing degeneration	Any	Ongoing degeneration	4th or bad	Low
E (excluded)	Degenerated	Any	Degenerated	5th or degenerated	None

### FF Samples

Following the oocyte retrieval, the FF samples were centrifuged at 4°C during 15 min at 1500 × *g*. The supernatant was filtered using 0.22-μm filter units (Merck KGaA, Darmstadt, Germany) to remove cellular debris, then aliquoted and stored at −20°C until use. Before and after centrifugation, an aliquot from 26 FF samples was used to determine hemoglobin (Hb) levels with a HemoCue Plasma/Low Hb System (Ängelholm, Sweden). This was done to ensure that the puncture procedure did not affect the Hb levels in the fluid, which, if present after the FF has been processed, can cause NO_2_ reduction or oxidation (Lundberg et al., 2008).

### NO_2_ and NO_3_ Measurements in FF

Nitrite and NO_3_ levels in FF samples were measured by HPLC-UV, using a method previously described by Croitoru (2012) with some changes, namely, in the column used, flowrate, duration, and without any derivatization step. In detail, the analysis was carried out on an Agilent 1100 Series HPLC System (Agilent Technologies, Santa Clara, CA, United States) equipped with a thermostated microwell plate autosampler, a quaternary pump, and a multiple wavelength absorbance detector. Standards and samples (40 μl) were injected into an Agilent Zorbax Eclipse XDB-C18 HPLC column (4.6 mm × 150 mm, 5 μm), thermostated at 25°C, and eluted at a flowrate of 400 μl/min during the whole separation. Mobile phase A consisted of 5 mM tetrabutylammonium hydroxide pH 2.5 (Sigma-Aldrich Química S.A., Madrid, Spain) and 8% *v/v* acetonitrile (VWR Chemicals, Barcelona, Spain) in water, while mobile phase B was methanol (VWR Chemicals, Barcelona, Spain). The gradient elution program was 100% solvent A for 10 min, a linear gradient from 0 to 50% solvent B for 20 min, and 10 min at constant 100% solvent B. The column was equilibrated with the starting composition of the mobile phase for 15 min before each analytical run. The 206 nm absorbance signal was recorded.

High-performance liquid chromatography standards were prepared in Milli-Q water using reagent grade sodium nitrite and sodium nitrate (Sigma-Aldrich Química S.A., Madrid, Spain). Both standards were prepared at a concentration of 1 mM, and serial dilutions from 100 to 0.1 μM were used to obtain the calibration curve for the analysis. FF samples were thawed and filtered through Amicon 3K centrifugal units (Merck KGaA, Darmstadt, Germany) to eliminate proteins. The centrifugal units were first rinsed with Milli-Q water to equilibrate the membrane and then centrifuged for 10 min at 14,000 × *g*. The receiver tube was replaced with a new one, and 400 μl of the sample was added to the centrifugal unit and centrifuged for 60 min at 14,000 × *g*. The clean filtrates were used for the analysis. The UV chromatograms at 206 nm from both standards and samples were analyzed with Chemstation Rev B.01.03.SR2 (Agilent Technologies, Santa Clara, CA, United States). The NO_2_ and NO_3_ peak areas in the standard solutions were used for the calculation of the calibration curve, from which the concentration in samples was obtained. The measurements were performed in duplicate, and the sum of mean values of NO_2_ and NO_3_ levels was used to calculate NOx concentration (Ratajczak-Wrona et al., 2013). The ratio between mean values of NO_3_ and NO_2_ levels was also determined.

### Statistical Analysis

Descriptive statistics were calculated for demographic, lifestyle, first donation cycle characteristics in the entire cohort, plus embryo production and quality by percentiles of NO_2_, NO_3_, NOx, and NO_3_/NO_2_ ratio. The presence of any associations was evaluated by using ANOVA and chi-square tests for continuous and categorical variables, respectively. These data were presented as mean (standard deviation, SD) or number of women (%). Multivariable mixed Poisson and logistic regression models with random slopes to account for repeated observations within a woman were used to compare total and MII oocyte yields, as well as the proportion of MII oocytes, across tertiles of NO_2_, NO_3_, NOx, and NO_3_/NO_2_ ratio. Categorical covariables were included using indicators for missing covariates when necessary. Multivariable-adjusted models included terms for age, body mass index, sleep time, coffee intake, smoking history, and leisure physical activity as potential confounders of the relation between NO metabolites and measures of ovarian response to hyperstimulation. Correlations (Spearman’s rho) between NOx levels and dietary pattern variables were also evaluated, namely, the Mediterranean diet score, the consumption of vegetables in general or specifically the ones with high NOx content like spinach. Tests for linear trend were conducted by modeling the tertiles of each metabolite, using the median analyte concentration values in each tertile, as a continuous linear term. For embryo development and quality, continuous variables were summarized by arithmetic mean, SD, range, and selected percentiles, including the median. Spearman’s rank correlation coefficients were used to explore the relationship between NO_2_, NO_3_, NOx, NO_3_/NO_2_ ratio, and embryonic parameters. All analyses were performed in SAS 9.4 (SAS Institute) and IBM SPSS 25.0 (IBM Corporation, Armonk, NY, United States).

## Results

**FIGURE 2:**
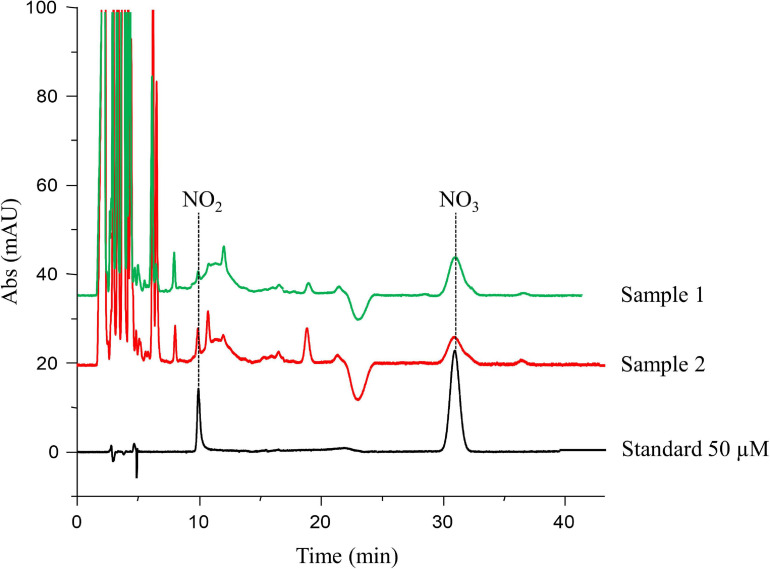
High-performance liquid chromatography (HPLC)-UV chromatogram. The peak located at 10.2 min was identified as nitrite (NO_2_), while the peak at 31.6 min was identified as nitrate (NO_3_). The peak areas of each compound in the standard solutions were used for the calculation of the calibration curve, from which the concentrations in follicular fluid samples (e.g., samples 1 and 2) were obtained.

Seventy-two women participating in the oocyte donation program at IVI-RMA Global Murcia (Spain), between February 2017 and September 2018, were included in our cohort. FF was obtained at oocyte retrieval in 93 oocyte donation cycles to measure NO_2_ and NO_3_ levels by HPLC-UV (Figure 2). The first peak, located at 10.2 min was identified as NO_2_, while the peak at 31.6 min was identified as NO_3_. When analyzing the chromatogram from different FF samples, we observed the same peaks with the same retention times (Figure 2).

The FF concentrations of NO_2_, NO_3_, NOx, and the NO_3_/NO_2_ ratio are reported in Table 2. NO_2_ levels ranged from 0.7 to 96.1 μM, NO_3_ levels ranged from 4.9 to 39.7 μM, NOx levels ranged from 5.6 to 109.5 μM, and NO_3_/NO_2_ ratio ranged from 0.1 to 31.5. NO_2_ and NO_3_ concentrations were unrelated to each other (*r* = −0.01). NO_2_ was positively correlated with NOx and negatively correlated with the NO_3_/NO_2_ ratio (Table 2).

**TABLE 2:** Follicular fluid levels of nitrite (NO_2_), nitrate (NO_3_), total nitric oxide (NOx), NO_3_/NO_2_ ratio, and Pearson correlation coefficients between these parameters.

				***R***			
	**Minimum**	**Maximum**	**Mean (SD)**	**NO_2_**	**NO_3_**	**NOx**	**NO_3_/NO_2_ ratio**
NO_2_	0.7	96.1	14.7 (12.3)	1.00	−0.01	0.89	−0.40
NO_3_	4.9	39.7	17.3 (6.3)		1.00	0.45	0.14
NOx	5.6	109.5	31.9 (13.7)			1.00	−0.29
NO_3_/NO_2_ ratio	0.1	31.5	2.6 (4.4)				1.00

*Minimum, maximum, and mean (standard deviation) concentrations of NO_2_, NO_3_, and NOx are expressed as μM. The values are representative of 93 follicular fluid samples.*

No significant differences were found when analyzing the following variables across tertiles of NO_2_ and NO_3_: age at egg donation, BMI, Mediterranean diet score, coffee and occasional alcohol intake, secondhand exposure to smoke, and sedentary behavior (Table 3). On the other hand, women with higher FF NO_2_ levels were more likely to sleep less [mean (SD) of 7.0 (2.0) h/day] and spend more time per week in leisure activities [mean (SD) of 3.7 (6.0) h/week]. Moreover, higher NO_2_ and NO_3_ concentrations were present in donors who smoked (22.2 and 23.6%, respectively), either at the time of the study or in the past. No significant correlations between NOx levels and vegetables intake were observed (Supplementary Tables 1, 2). The characteristics of the first donation cycle, such as the number of stimulation days, total dose of FSH, and the oocyte fate, were similar across tertiles of NO_2_ and NO_3_, but donors with low FF NO_2_ levels likely had a higher E2 peak [mean (SD) of 2004.2 (1140.8) pg/mL].

**TABLE 3:** Demographic, lifestyle, and first cycle characteristics by nitrite (NO_2_) and nitrate (NO_3_) tertiles of healthy women undergoing ovarian stimulation as part of their participation in an oocyte donation program (*n* = 72).

	**NO_2_**				**NO_3_**			
	**1st tertile, *n* = 25**	**2nd tertile, *n* = 23**	**3rd tertile, *n* = 24**	***p* value^2^**	**1st tertile, *n* = 24**	**2nd tertile, *n*** = **24**	**3rd tertile, *n* = 24**	***p* value^2^**
***Demographic and lifestyle*^1^**								
Age at egg donation, years	24.9 (4.9)	23.7 (3.9)	24.6 (4.0)	0.83	23.6 (4.2)	24.6 (4.2)	25.1 (4.4)	0.22
Body mass index, kg/m^2^	22.4 (3.1)	22.7 (2.6)	23.2 (3.3)	0.35	23.6 (3.1)	22.3 (2.9)	22.3 (2.9)	0.12
Sleep time/day, h	8.7 (1.9)	8.6 (1.1)	7.0 (2.0)	0.01	7.6 (2.5)	8.1 (2.1)	8.2 (1.2)	0.45
Mediterranean diet score (Panagiotakos et al., 2007)	34.1 (6.1)	31.6 (6.8)	32.6 (7.5)	0.53	31.2 (7.6)	35.7 (5.6)	32.0 (6.5)	0.80
Coffee intake, servings/week	4.2 (10.1)	2.2 (3.0)	8.2 (9.0)	0.15	4.7 (8.5)	3.8 (6.2)	6.7 (10.1)	0.46
Occasional alcohol intake, N (%)	14 (19.4)	12 (16.7)	12 (16.7)	0.91	11 (15.3)	13 (18.1)	14 (19.4)	0.68
Ever smoker, N (%)	15 (20.8)	5 (6.9)	16 (22.2)	0.00	11 (15.3)	8 (11.1)	17 (23.6)	0.03
Self-reported exposure to second hand smoke, N (%)	6 (11.3)	6 (11.3)	6 (11.3)	0.93	8 (15.1)	4 (7.6)	6 (11.3)	0.37
Leisure moderate/vigorous activity, h/week	0.7 (1.8)	1.4 (2.8)	3.7 (6.0)	0.04	2.4 (2.9)	1.2 (2.6)	2.2 (5.8)	0.96
Sedentary behavior, h/week	25.4 (28.4)	23.2 (11.6)	30.3 (21.6)	0.52	28.0 (21.2)	23.2 (28.0)	28.0 (16.4)	0.95
***First cycle characteristics^1^***								
Number of stimulation days, N	9.9 (1.8)	9.8 (1.1)	9.7 (1.1)	0.76	9.7 (1.2)	9.5 (1.0)	10.2 (1.8)	0.32
E2 peak, pg/mL	2004.2 (1140.8)	1474.7 (668.5)	1328.1 (667.1)	0.02	1619.4 (726.6)	1748.6 (1275.1)	1535.3 (678.1)	0.78
FSH total dose, IU/L	1450.5 (395.0)	1413.3 (531.0)	1649.6 (453.1)	0.19	1398.1 (570.7)	1516.5 (352.3)	1604.2 (429.5)	0.19
Oocyte fate, N (%)				0.65				0.58
Fresh transfer	12 (22.6)	6 (11.3)	7 (13.2)		7 (13.2)	8 (15.1)	10 (18.9)	
Vitrified	3 (5.7)	1 (1.9)	3 (5.7)		4 (7.6)	2 (3.8)	1 (1.9)	
Mixed	6 (11.3)	7 (13.2)	8 (15.1)		6 (11.3)	8 (15.1)	7 (13.2)	

*^1^Data are presented as mean (standard deviation) or number of women (%). ^2^Differences across categories were tested using an ANOVA test for continuous variables and a chi-square test for categorical variables.*

Follicular fluid NO_2_ and NO_3_ were unrelated to total or mature oocyte yield (Table 4). The multivariable-adjusted MII yield (95% CI) for women in the lowest and highest tertiles of NO_2_ was 12.4 (10.2, 15.1) and 13.2 (10.9, 16.0) (p, linear trend = 0.38) and 14.1 (11.7, 17.1) and 12.2 (9.9, 15.0) for NO_3_ (p, linear trend = 0.14), respectively. When MII oocytes were considered as the proportion of total oocytes, however, the proportion of MII oocytes increased with increasing FF NO_2_ levels but decreased with increasing NO_3_ levels. The adjusted proportion (95% CI) of MII oocytes for women in the lowest and highest FF levels of NO_2_ were 68% (58–77%) and 79% (70–85%) (p, linear trend = 0.02), whereas the proportion of MII oocytes for women in extreme tertiles of FF NO_3_ levels were 79% (70–85%) and 68% (57–77%) (p, linear trend = 0.03). NOx and the NO_3_/NO_2_ ratio were unrelated to the total and mature oocyte yield, whether expressed in absolute or relative terms (Table 4). Summary statistics for embryo development and quality are shown in Table 5.

**TABLE 4:** Association between nitric-oxide-related parameters and the adjusted oocyte yield, number, and proportion of MII oocytes in of healthy women undergoing ovarian stimulation as part of their participation in an oocyte donation program.

	**n cycles/n donors**	**Oocyte yield, n^1^**	**MII oocytes, n^1^**	**MII oocyte proportion (%)^2^**
**NO_2_**	93/72			
1st tertile	31/25	18.2 (15.0, 22.1)	12.4 (10.2, 15.1)	68 (58, 77)
2nd tertile	31/23	18.5 (15.1, 22.6)	14.0 (11.4, 17.1)	78 (69, 85)
3rd tertile	31/24	18.3 (15.1, 22.1)	13.2 (10.9, 16.0)	79 (70, 85)
p, linear trend^3^		0.94	0.38	0.02
**NO_3_**	93/72			
1st tertile	30/24	18.5 (15.4, 22.4)	14.1 (11.7, 17.1)	79 (70, 85)
2nd tertile	32/24	18.1 (15.0, 21.9)	13.2 (10.9, 16.1)	78 (70, 85)
3rd tertile	31/24	18.3 (15.0, 22.5)	12.2 (9.9, 15.0)	68 (57, 77)
p, linear trend^3^		0.88	0.14	0.03
**NOx**	93/72			
1st tertile	30/26	19.2 (16.0, 23.1)	13.7 (11.4, 16.5)	74 (65, 81)
2nd tertile	31/20	21.2 (17.3, 25.8)	15.2 (12.4, 18.6)	77 (68, 84)
3rd tertile	32/26	16.4 (13.7, 19.7)	12.2 (10.1, 14.6)	77 (69, 84)
p, linear trend^3^		0.14	0.31	0.41
**NO_3_/NO_2_ ratio**	93/72			
1st tertile	31/23	21.7 (16.7, 28.1)	14.1 (10.7, 18.6)	68 (52, 80)
2nd tertile	31/23	16.8 (13.8, 20.6)	12.9 (10.4, 15.9)	82 (73, 88)
3rd tertile	31/26	17.2 (13.4, 22.2)	12.6 (9.6, 16.5)	74 (61, 84)
p, linear trend^3^		0.18	0.54	0.42

*NO_2_, nitrite; NO_3_, nitrate; NOx, nitric oxide. Models were run using ^1^Poisson regression with log link and ^2^binomial regression with logit link. All data are presented as least square means (95% CI), adjusted for age, body mass index, sleep time, coffee intake, smoking history, and leisure physical activity. ^3^p, linear trend was calculated by modeling the tertiles of each metabolite, using the median analyte concentration values in each tertile as a continuous linear term.*

**TABLE 5:** Summary statistics for embryo development and quality from oocytes of healthy women undergoing ovarian stimulation as part of their participation in an oocyte donation program (*n* = 75).

	**Mean (SD)**	**Minimum**	**Selected percentiles**					**Maximum**
			**5th**	**25th**	**50th**	**75th**	**95th**	
Number of inseminated oocytes	13.8 (5.1)	6	7.8	10	13	16	24	37
Fertilization rate (%)	72.7 (16.9)	16.7	36.7	62.5	73.3	85.7	100	100
Number of embryos obtained (cleaved)	10.1 (4.5)	1	4.8	7	9	12	21.4	24
Embryo rate (%) (cleaved)	61.8 (29.6)	0	8.9	39.1	66.7	87.5	100	100
Number of embryos on day 5	6.4 (4.6)	0	0,8	3	6	8	17	24
Embryos A + B	3.8 (2.8)	0	0	2	3	5	9	14
Embryos C	0.9 (1.2)	0	<1	<1	<1	1	3.2	5
Embryos D	1.2 (1.6)	0	<1	<1	1	2	5	8

*Embryo quality was classified according to the Spanish Association for the Study of the Biology of Reproduction (ASEBIR), which established the potential of embryo implantation as: A, maximum; B, high; C, medium; D, low (see Table 1).*

**FIGURE 3:**
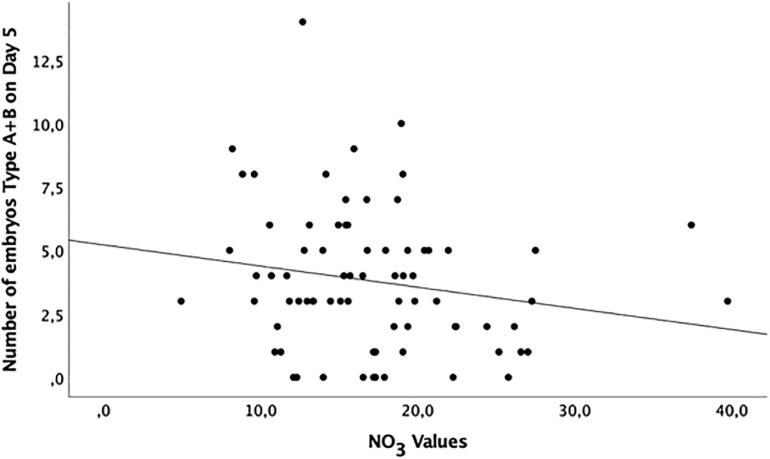
Distribution of grade A and B embryos (maximum and high potential of implantation, respectively) at day 5 of development according to the NO_3_ levels in the follicular fluid of healthy women undergoing ovarian stimulation as part of their participation in an oocyte donation program (*n* = 75).

A suggestive (borderline) inverse correlation between NO_3_ levels and the number of embryos type A + B on day 5 (*p* = 0.07) and embryo cleavage rate on day 5 (*p* = 0.08) were observed (Figure 3 and Table 6).

**TABLE 6:** Correlation between NO_2_, NO_3_, NOx, ratio NO_3_/NO_2_, and embryo development and quality of healthy women undergoing ovarian stimulation as part of their participation in an oocyte donation program (*n* = 75).

	**NO_2_**		**NO_3_**		**NOx**		**Ratio NO_3_/NO_2_**	
	***r***	***p* value**	***r***	***p* value**	***r***	***p* value**	***r***	***p* value**
Number of inseminated oocytes	0.05	0.68	−0.12	0.29	−0.01	0.92	−0.07	0.57
Fertilization rate (%)	0.13	0.25	−0.02	0.84	0.11	0.34	−0.17	0.16
Number of embryos obtained (cleaved)	0.06	0.61	−0.11	0.35	0.02	0.88	−0.11	0.33
Embryo rate (%) (cleaved)	−0.05	0.65	−0.20	0.08	−0.18	0.11	−0.02	0.87
Number of embryos on day 5	0.01	0.91	−0.19	0.10	−0.11	0.33	−0.08	0.49
Embryos A + B	0.08	0.50	−0.20	0.07	−0.08	0.51	−0.15	0.29
Embryos C	−0.05	0.65	−0.11	0.37	−0.08	0.48	0.02	0.86
Embryos D	−0.02	0.88	0.03	0.81	0.02	0.90	0.06	0.62

*Embryo quality was classified according to the Spanish Association for the Study of the Biology of Reproduction (ASEBIR), which established the potential of embryo implantation as: A, maximum; B, high; C, medium; D, low (see Table 1). r, Spearman’s rank correlation coefficient.*

## Discussion

One of the factors associated with successful pregnancy during IVF is oocyte quality (Lee et al., 2000). The oocyte development takes place in a dynamic microenvironment, where the complex composition of the FF is very important (Vignini et al., 2008). Among other molecules, FF contains free radicals, like NO, which is actively synthesized by the granulosa cells (Lee et al., 2000). This means that NO and/or its by-products may be potential biomarkers for IVF outcome, and several studies tested this hypothesis in patients undergoing fertility treatment (Barroso et al., 1999; Barrionuevo et al., 2000; Lee et al., 2000; Battaglia et al., 2006; Vignini et al., 2008; Zhao et al., 2010; Goud et al., 2014).

The assessment of NO concentration can be performed indirectly by assaying two anions, i.e., NO_2_ and NO_3_ (Revelli et al., 2009). In the present work, we described how a previously validated technique for measuring simultaneously these ions (Croitoru, 2012) can also be applied in FF samples.

The levels of NO_2_ and NO_3_, described in our cohort, did not predict the total number of oocytes recovered from the donors or the MII oocytes count. Other studies described similar findings. Yalçınkaya et al. (2013) reported the absence of a correlation when evaluating the relationship between NO and IVF parameters such as the number of mature oocytes, fertilization rate, and embryo grading. Lee et al. (2000) found no significant differences between FF NO levels and the maturity and quality of the oocyte. Moreover, the NO_2_/NO_3_ concentrations measured in both serum and FF were not good markers of ovarian response or pregnancy in IVF cycles (Manau et al., 2000).

Interestingly, our data suggest that the FF concentrations of NO_2_ and NO_3_ are associated with the proportion of MII oocytes; particularly, the latter was related directly to NO_2_ levels and inversely to NO_3_ levels. Even though these stable ions derive from NO, previous research reports evidenced that they can be physiologically recycled to form again NO (Lundberg et al., 2008), likely to be employed for protein S-nitrosylation during maturation process of the oocyte (Romero-Aguirregomezcorta et al., 2014). According to our results, FF NO_2_ levels appear to be more representative for NO formation, as shown by the Pearson correlation coefficients. The formation of NO_2_ takes place, for instance, by NO auto-oxidation, NO_3_ reduction (Lundberg et al., 2008), or through a reaction catalyzed by the multicopper oxidase ceruloplasmin (Shiva et al., 2006), which is also a FF component (Suchanek et al., 1990; Gonzalès et al., 1992; Gentry et al., 2000). It has been shown that its levels depend on the ovarian stimulation protocol (Suchanek et al., 1990), and it was described as an indicator of oocyte maturation, since the ceruloplasmin concentration was higher in follicles containing a mature egg (Gulamali-Majid et al., 1987) and that later underwent cleavage (Gonzalès et al., 1992). NO_2_ formation via ceruloplasmin also produces nitrous acid (Shiva et al., 2006). Both these species can be converted back into NO in the presence of ascorbate (reviewed by Lundberg et al., 2008), which has been identified in the FF (Cigliano et al., 2002; Khan and Das, 2011).

On the other hand, our work suggests an inverse relation between NO_3_ levels in FF and the potential of embryos to implant in the uterus. Higher FF NO_3_ levels were found to be consistent with the presence of nitrotyrosine in granulosa cells, which is indicative of peroxynitrite synthesis (Goud et al., 2014). The synthesis of peroxynitrite takes place when NO interacts with the superoxide anion (Burton and Jauniaux, 2011). The latter species is physiologically produced during folliculogenesis, but lifestyle factors can lead to an unbalance in its regulation (Agarwal et al., 2012). In turn, the peroxynitrite causes lipid peroxidation and cellular damage (Radi et al., 1991). Goud et al. (2014) reported increased FF NO_3_ levels in women with versus without endometriosis. Additionally, the affected women who achieved a pregnancy had significantly lower FF NO_3_ levels. The authors, therefore, suggested that the presence of high concentrations of NO_3_ and peroxynitrite in the oocyte microenvironment may contribute to a poor follicular health, oocyte quality, embryo quality, and potential embryo implantation. This might justify the negative correlation between the proportion of MII oocytes and embryo quality and the NO_3_ levels reported in our study.

In conclusion, the direct measurement of NO in biological fluids is problematic because of its short-lived nature. Nonetheless, different techniques can be used to determine NO_2_ and NO_3_ levels, but in some cases, it is not possible to simultaneously detect these ions, and complex derivatization procedures might be involved. HPLC-UV represents a valid alternative, as it is a rapid, sensitive, and selective method to detect NO_2_ and NO_3_, besides from having already been used in plant and animal samples. In this study, we successfully tested this method with human FF samples, which allowed us to investigate how the NO_2_ and NO_3_ levels in this fluid correlate with the ovarian response and embryo quality in human oocyte donors. We detected an association between the FF levels of these species and the proportion of MII oocytes and, possibly, with the quality and implantation potential of embryos derived from those oocytes. However, we have not detected an association between NO_2_ and NO_3_ levels in FF and the total and MII oocyte yield. This absence of a correlation with total oocyte counts may reflect a lack of association of the NO_2_/NO_3_-mediated pathway with the ovarian reserve. Although there is no correlation between NO_2_ and NO_3_ with the ovarian reserve, their levels could indicate the maturation of the oocytes (NO_2_ above all) and embryo quality (NO_3_ above all), and both NO_2_ and NO_3_ are representative for the formation of NO. It is unclear to what extent differences in MII oocyte proportion and embryo quality due to different FF levels of NO_2_ or NO_3_ could impact downstream outcomes like pregnancy and birth rates. Further studies should address these questions in the patients who received the oocytes obtained from our cohort of donors.

## Data Availability Statement

The raw data supporting the conclusions of this article will be made available by the authors, without undue reservation.

## Ethics Statement

The studies involving human participants were reviewed and approved by the Ethics Review Committee of CEIC Hospital General Universitario Jose Maria Morales Meseguer (Murcia, Spain) (Approval No. EST: 06/17) and registered at https://clinicaltrials.gov/ (ID: NCT03307655). Women participating in the gamete donation program at IVI-RMA Global (Murcia, Spain) were invited to participate in the study. All the donors who accepted provided their written informed consent. The patients/participants provided their written informed consent to participate in this study.

## Author Contributions

F-DS recruited the oocyte donors, analyzed the data, and wrote the manuscript. AC-G recruited the oocyte donors, analyzed the data, and reviewed the manuscript. CS-Ú and EA analyzed the data and wrote the manuscript. JM-S recruited the oocyte donors, provided the clinical and lifestyle-related data, and reviewed the manuscript. JC designed the statistical analysis, analyzed the data, and reviewed the manuscript. CM conceived and designed the study and reviewed the manuscript. All authors read and approved the final manuscript.

## Conflict of Interest

The authors declare that the research was conducted in the absence of any commercial or financial relationships that could be construed as a potential conflict of interest.
