# Supplementary material for: Nitrite and Nitrate Levels in Follicular Fluid From Human Oocyte Donors Are Related to Ovarian Response and Embryo Quality
Source: Front Cell Dev Biol. 2021 Apr 14;9:647002. doi: 10.3389/fcell.2021.647002 (PMC8079729; doi:10.3389/fcell.2021.647002)
Supplement: Supplementary file 1 [file Data_Sheet_1.pdf]

Supplementary Material

Supplementary Table 1. Summary statistics for intake of vegetables and other items presumed to be close to Mediterranean dietary pattern. Oocyte donors (n=43) registered their frequency of several food items intake per week, including the spinach, a previously described vegetable with high mean nitrate content (Lidder and Webb, 2013), a general vegetables group as the sum of different food items (cabbage, cauliflower, broccoli, cooked or raw, artichokes, asparagus, carrot, spinach or cooked chard, lettuce, endives, endive, onion, cooked green beans, aubergines, zucchini, cucumbers, peppers, boiled corn, and tomato), and the adherence to the Mediterranean diet as the sum of the scores corresponding to the intake of relevant groups of food items (non-refined cereals, fruits, vegetables, legumes, olive oil, fish, and potatoes) according with Panagiotakos et al. (2007).

	**Mean**	**Standard Deviation**	**Selected Percentiles**		
			**25th**	**50th**	**75th**
**High nitrate vegetable** (servings/week)	1.3	3.1	0.0	0.5	1.0
**Vegetables group score** (0-5)	3.5	1.4	2.0	4.0	5.0
**Mediterranean diet score** (0-55)	32.7	5.7	30.0	34.0	35.0

High nitrate vegetable: mean frequency (servings/week) of spinach intake per week (a known vegetable abundant in nitrate content). Vegetables group: mean frequency of vegetable group score intake (0-5: never, rare, frequent, very frequent, weekly, and daily, respectively). Mediterranean diet score: diet score (0-55) of adherence to the Mediterranean diet (Panagiotakos et al., 2007).

Supplementary Table 2. Correlations between NOx in follicular fluid and vegetables intake in oocyte donors (n=43).

	**NO_2**		**NO_3**		**NOx**		**Ratio NO_3/NO_2**	
	r	p-value	r	p-value	r	p-value	r	p-value
**High nitrate vegetable**	-0.080	0.61	0.097	0.54	-0.104	0.51	0.078	0.62
**Vegetable group**	-0.082	0.60	-0.042	0.79	-0.133	0.39	0.047	0.77
**Mediterranean diet score**	-0.033	0.83	-0.040	0.80	-0.118	0.45	0.029	0.85

r: Spearman's rank correlation coefficient (Spearmans's rho).
